# Icariin against osteoporosis: a review of advances in molecular mechanisms to biomedical applications

**DOI:** 10.3389/fphar.2025.1688847

**Published:** 2025-11-25

**Authors:** Boyi Fu, Shasha Yu, Shengxiong Chen, Bo Hu

**Affiliations:** 1 School of Sports Medicine and Health, Chengdu Sport University, Chengdu, China; 2 Shenzhen Prevention and Treatment Center for Occupational Diseases, Shenzhen, China; 3 School of Life Science, Beijing University of Chinese Medicine, Beijing, China

**Keywords:** icariin, osteoporosis, bone metabolism, bone homeostasis, drug delivery, molecular mechanisms, animal models, therapeutic application

## Abstract

Osteoporosis (OP) is characterized by decreased bone mass and deterioration of bone microstructure, significantly increasing fracture risk. Icariin (ICA), a natural compound, has demonstrated efficacy in improving bone microstructure and bone mineral density (BMD) across multiple OP models, with its targeting efficacy enhanced through innovative drug delivery systems. This review systematically summarizes recent advances in ICA research, focusing on its application dosage forms, therapeutic performance in various animal models, and underlying molecular mechanisms. In order to ensure a comprehensive and reliable report, we conducted a systematic search in the core collection of web of science according to PRISMA guidelines, and finally included 182 publications for in-depth analysis. ICA’s therapeutic efficacy is enhanced through innovative delivery systems, including traditional Chinese medicine formulations and advanced biomaterials. Studies across postmenopausal, glucocorticoid-induced, aging, and diabetic OP models consistently demonstrate ICA’s ability to improve bone microarchitecture and BMD. Mechanistically, ICA exerts dual-regulation effects by promoting osteogenesis while inhibiting osteoclastogenesis, coupled with multi-target actions involving autophagy regulation, anti-inflammatory effects, iron overload mitigation, and oxidative stress reduction. In conclusion, ICA’s comprehensive and multi-mechanistic intervention strategy, augmented by advanced delivery systems, presents a natural, safe, and efficacious candidate for OP treatment. This review synthesizes critical advances from molecular mechanisms to biomedical applications, supporting further clinical translation of ICA-based therapies.

## 1 Introduction

Osteoporosis (OP), characterized by reduced bone mass and microstructural deterioration, is a systemic bone metabolic disease. Prevalent among postmenopausal women and the elderly, it significantly increases fracture risk and poses a global public health challenge (Ilyas et al., 2024; Tan et al., 2024; Chaichit et al., 2022; Lin et al., 2020; García-Espinosa et al., 2024). At present, there are mainly three kinds of drugs for the prevention or treatment of OP in clinic: inhibitors of bone resorption (such as bisphosphonates, RANKL inhibitors, estrogen and related drugs, *etc.*), which can slow down bone loss by inhibiting the activity of osteoclasts; Osteogenic drugs (such as parathyroid hormone analogue teriparatide) can increase bone mass and strength by stimulating osteoblasts (Su et al., 2024; Khoury, 2024); Drugs with dual effects (such as sclerostin inhibitor Romozozumab and strontium ranelate) can simultaneously inhibit bone resorption and promote bone formation (Bonnelye et al., 2008). However, the long-term safety and potential adverse reactions of existing drugs are important considerations in their clinical application. For example, bisphosphonates can effectively inhibit bone resorption, but there is a risk of jaw necrosis after long-term use (Park et al., 2025). Strontium ranelate has been proved to effectively reduce the risk of vertebral and hip fractures in postmenopausal osteoporosis patients, but it also increases the risk of venous thromboembolism, myocardial infarction and severe skin allergic reactions (Cacoub et al., 2013). Romosozumab demonstrates significant efficacy in increasing bone mineral density; however, its clinical application is limited by the high cost and potential cardiovascular risk (Takeuchi, 2025). These limitations highlight the continuing demand for safer, more effective, and more economical treatments in the field of osteoporosis treatment.

In contrast, icariin (ICA), as a natural anti osteoporosis drug candidate, has shown unique appeal. ICA, the primary active flavonoid glycoside in *Epimedium* spp., has been used in traditional Chinese medicine (TCM) for thousands of years, which provides strong support for its good safety characteristics (Wang et al., 2024; Quan et al., 2024). First of all, in terms of therapeutic mechanism, ICA has a significant dual regulatory effect: it can not only promote bone formation by activating Hippo Yap/TAZ (Lin et al., 2024), IGF-1/ER α (Zhou et al., 2021) and other signaling pathways, but also inhibit bone resorption by inhibiting nuclear factor-κB ligand (RANKL)-induced osteoclast differentiation (Ji et al., 2022; Cheng et al., 2022). Its core advantage is that it can effectively regulate the imbalance of bone homeostasis through multi-target and multi-channel synergy (e.g., targeting PPARγ, ERα/AKT/β-Catenin) (Long et al., 2022; Cheng et al., 2024). This “network” treatment strategy may be more suitable for the treatment of complex chronic diseases such as osteoporosis than single target powerful drugs. Secondly, at the clinical and economic levels, numerous clinical trials support the efficacy of its compound preparations against primary/secondary osteoporosis (Wu L. et al., 2017; Wang et al., 2016). In addition, as a small molecule natural product, ICA has lower potential production cost, especially when compared with expensive biological agents such as Romozozumab, this advantage is more prominent, and is expected to become an economic and universal treatment option. Therefore, in-depth study of ICA has important strategic significance for the development of safe, effective and economical new anti osteoporosis drugs.

**FIGURE 1:**
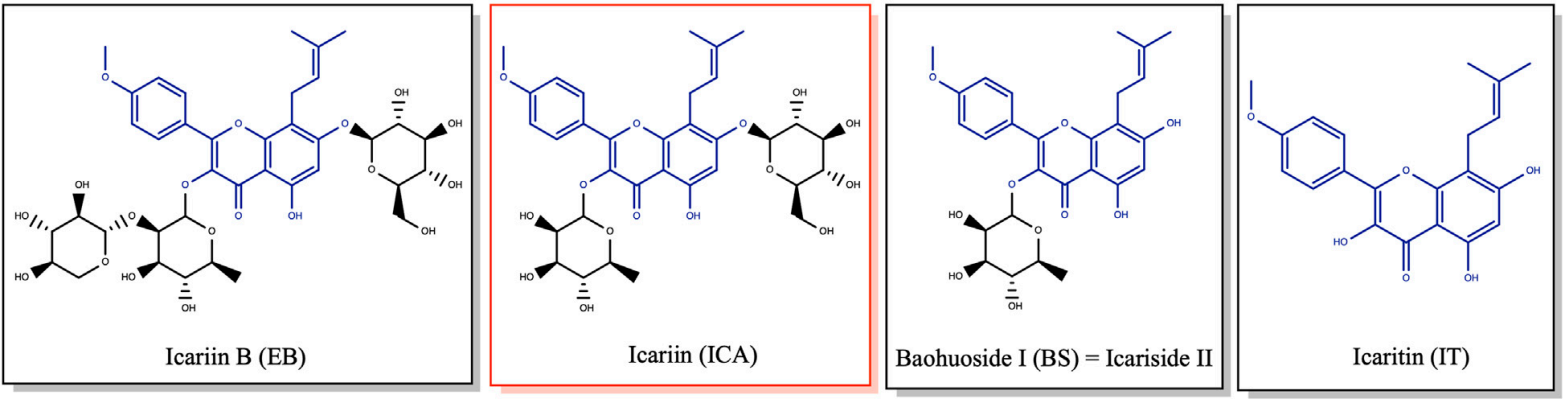
Icariin, its analogues and main metabolites.

Building upon these advantages of ICA, substantial research efforts have been dedicated to addressing its pharmacokinetic limitations to facilitate clinical translation. At the application level, innovative delivery systems have significantly enhanced ICA’s efficacy and targeting to address bioavailability challenges (Teo et al., 2019; Xin et al., 2015). Compound traditional Chinese patent medicines and simple preparations (Wang et al., 2025; Wang et al., 2024) can improve efficacy through multi-component synergism (Wang et al., 2025; Ren L. et al., 2023). Innovative biomaterial delivery systems [e.g., exosomes (Han et al., 2025), composite hydrogels (Zhang et al., 2025; Li et al., 2024a; Wu Y. et al., 2025), bioglass scaffolds (Kong et al., 2025)] substantially increase ICA’s targeting and efficacy. Numerous studies in various animal models—including postmenopausal (Chen C. et al., 2023), glucocorticoid-induced (Zhang L. et al., 2021; Zhang et al., 2015), and aging (Xu et al., 2018) rodent models, as well as chicken (Huang J. et al., 2020) and fish models (Jiang J. et al., 2022)—have reported ICA’s treatment of osteoporosis. These studies demonstrate ICA’s therapeutic efficacy from multiple perspectives and provide important references for developing drug treatments for different osteoporosis types. At the molecular level, ICA exerts central effects by synergistically activating osteogenic pathways [e.g., SPI1/SMAD5 (Zhang J. et al., 2024), Wnt/β-catenin (Gao et al., 2021)] and inhibiting osteoclastogenesis [e.g., Cullin 3/Nrf2 (Si et al., 2024), RANKL-p38/ERK-NFAT (Cheng et al., 2022)]. Key mechanisms include: upregulating osteogenic gene expression in bone marrow stromal cells (BMSCs); suppressing adipogenic differentiation markers [e.g., PPARγ (Chen X. et al., 2025)] and oxidative stress factors [NOX1/NOX4 (Ji et al., 2022)]; restoring OPG/RANKL balance to inhibit osteoclastogenesis (Sun et al., 2021); and optimizing the bone microenvironment *via* lipid metabolism reprogramming (Sharifi et al., 2020). ICA also activates autophagy flux to inhibit pro-apoptotic factors (Li et al., 2024b), suppresses the NF-κB inflammatory pathway (Zhang et al., 2014), chelates free iron ions to alleviate iron overload-induced oxidative damage (Tian et al., 2016), and enhances bone cell antioxidant capacity through Nrf2 pathway activation (Zhang C. et al., 2023), collectively protecting bone cell function. Its active metabolites, as shown in Figure 1, target mesenchymal stem cell bone morphogenesis pathways, underscoring multi-target advantages (Indran et al., 2016).

**FIGURE 2:**
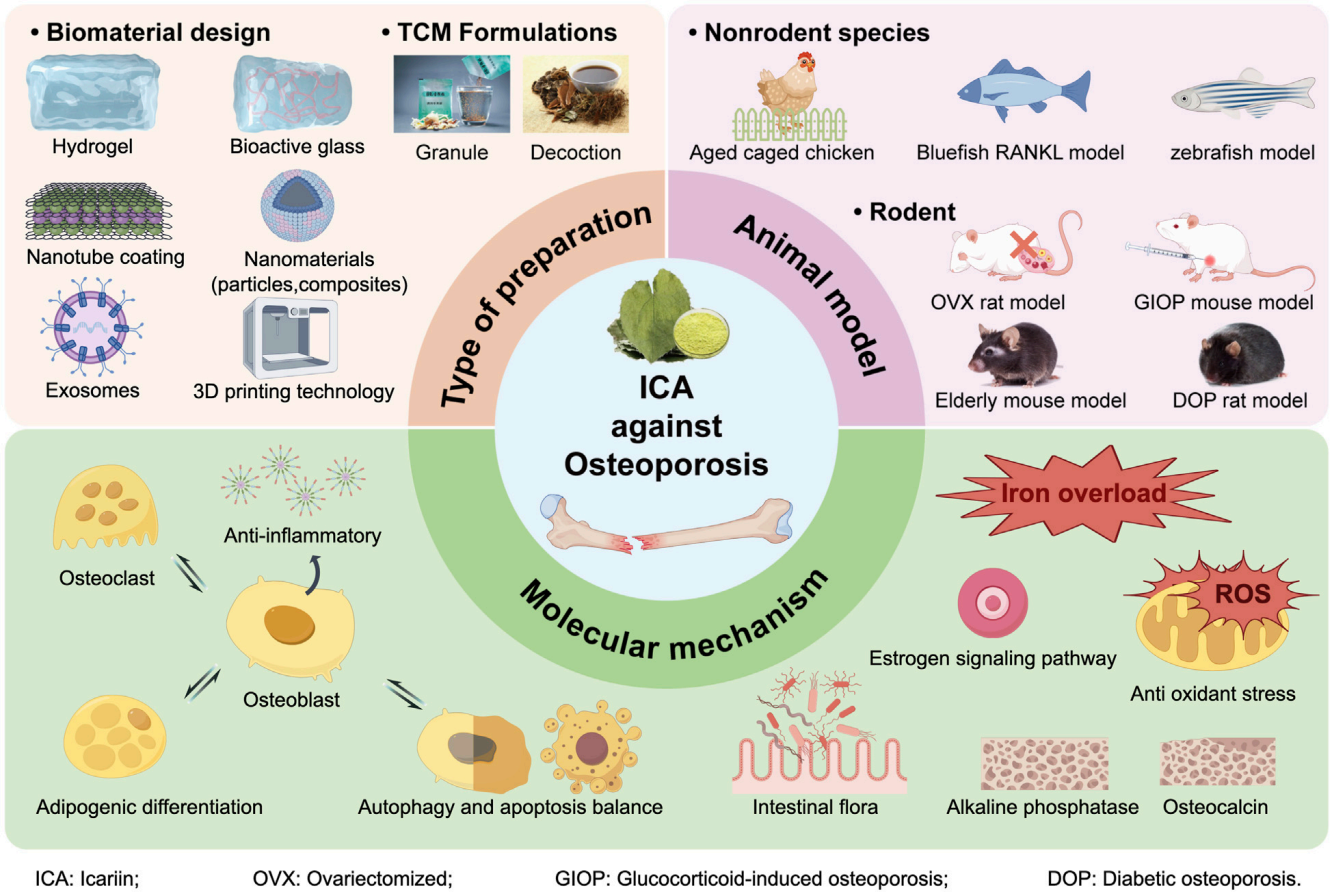
Photograph abstract: Application dosage form, animal model and molecular mechanism of icariin for the treatment of osteoporosis.

This review systematically integrates ICA’s anti-OP research progress (Figure 2), summarizes its dosage forms—from traditional/simple preparations to novel biomaterial delivery systems—and collates therapeutic efficacy across osteoporosis animal models. Finally, it comprehensively examines ICA’s multi-target mechanisms, spanning bidirectional regulation of osteogenesis/osteoclast inhibition, autophagy/apoptosis balance, anti-inflammation, iron overload mitigation, antioxidant stress, and metabolic homeostasis. The review aims to provide theoretical support for translating ICA from basic research to clinical practice.

## 2 Review methodology

**FIGURE 3:**
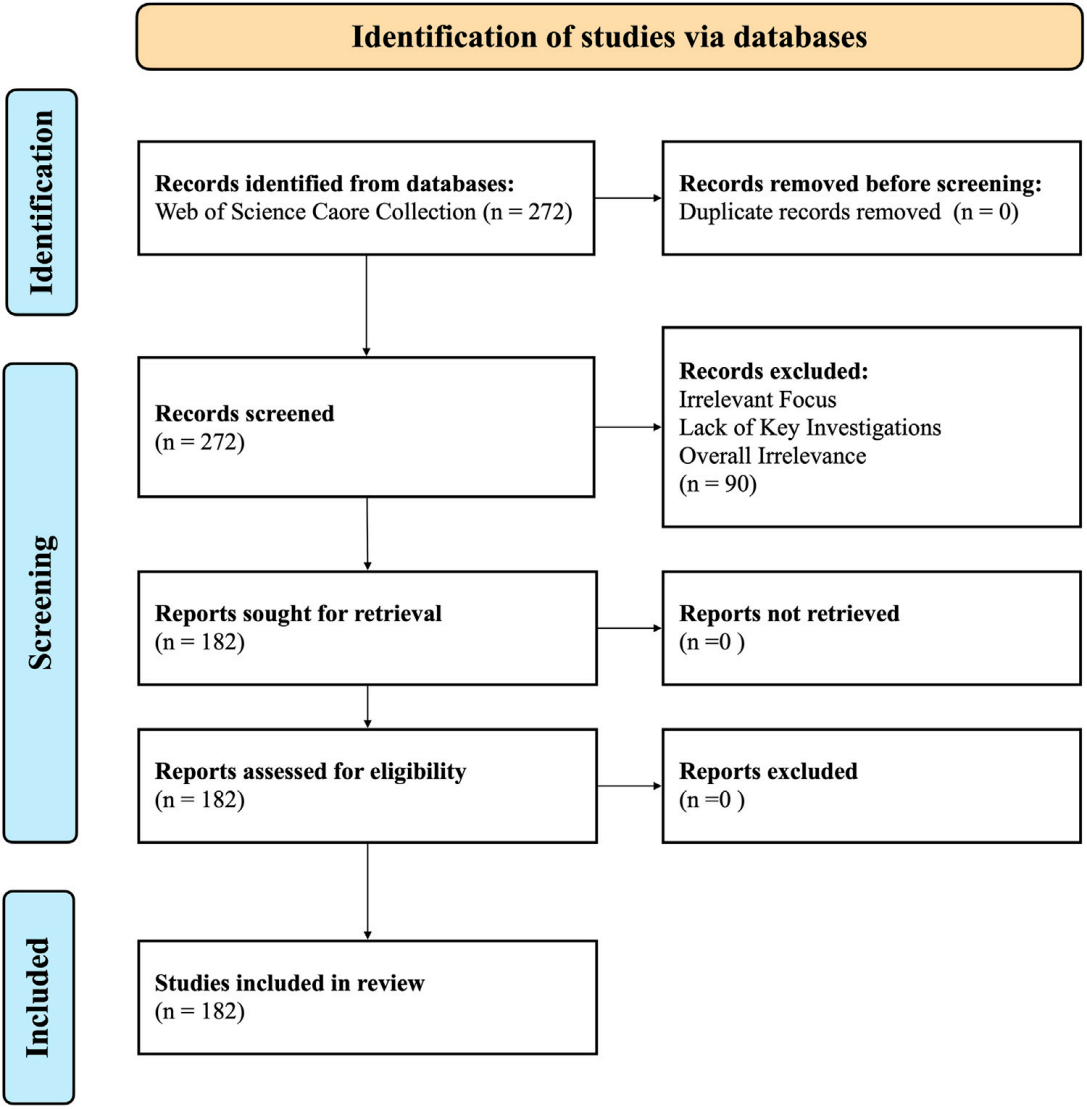
PRISMA flowchart of the literature screening.

The literature search for this review was conducted exclusively in the Web of Science Core Collection. This database was selected to ensure the inclusion of a consistent and high-quality dataset comprised of peer-reviewed articles from rigorously indexed journals, thereby enhancing the reliability and scholarly rigor of our analysis. The search, which encompassed literature published up to May 2025, was structured using a combination of topic-specific keywords. The search strategy was as follows: #1 (Osteoporosis Concept): TS=[(osteoporosis) OR (Osteoporoses) OR (Bone Loss, Age-Related)]. #2 (Icariin Concept): TS=((icariin) OR (3-((6-deoxymannopyranosyl)oxy)-7-(glucopyranosyloxy)-5-hydroxy-2-(4-methoxyphenyl)-8-(3-methyl-2-butenyl)-4H-1-benzopyran-4-one)). Final Search: #1 AND #2 (Initial Results: 272). The screening process involved a two-tiered approach. First, the titles and abstracts of the 272 identified records were reviewed. This led to the exclusion of 90 publications that were deemed irrelevant. Primary reasons for exclusion were studies not focusing on icariin’s anti-osteoporotic effects, or those lacking investigations into dosage forms, mechanisms of action, or animal models. Subsequently, a full-text assessment of the remaining 182 articles was performed to confirm their eligibility, resulting in the final inclusion of n = 182 publications for in-depth analysis in this systematic review. This review strictly followed the systematic evaluation specifications of the PRISMA guidelines, and the specific literature screening process is detailed in the visualisation flowchart shown in Figure 3.

## 3 Progress in the application of icariin dosage forms

### 3.1 Traditional Chinese medicine formulations

Icariin (ICA), the core component of Epimedium in TCM (Shen et al., 2007; Sze et al., 2010), faces clinical translation challenges due to its low bioavailability and complex mechanism. Current research focuses on two primary approaches: 1) Enhancing bone density and regulating bone metabolism through herbal formulation; 2) Developing novel dosage forms combined with omics technology to elucidate mechanisms regulating lipid metabolism, calcium balance, and signaling pathways, paving the way for precise treatment (as shown in Table 1).

**TABLE 1:** Progress of icariin formulations for the treatment of osteoporosis.

Large category	Classification	Specific content	Effect	References
Traditional Chinese patent medicines and simple preparations granules/decoction	Traditional Chinese patent medicines and simple preparations	Gu Shu Kang	Inhibit bone resorption	Wang et al. (2024), Li et al. (2022)
		Kunxinning	Relieve bone loss	Wang et al. (2025)
		Xianling gubao capsule	Improve bone mineral density	Zhu et al. (2012)
		Gushu Dan		Feng et al. (2022)
	Herbal decoction	Er-xian decoction	Promote bone formation and inhibit bone resorption	Qin et al. (2008), Ren L. et al. (2023)
Biomaterial	Hydrogel	Simulate the characteristics of bone matrix	Repair bone defects	Zhang et al. (2025)
	3D Printing	Bioactive glass	Activate osteogenesis and inhibit osteoclastogenesis	Kong et al. (2025)
		Electronic jet printing		
		Titanium alloy bracket	Enhance bone integration	Wang S. et al. (2022)
	Nanomaterials	Selenium gold shell	Inhibit inflammation	Li et al. (2024a)
		Nanoparticles	Promote osteogenesis	Yan et al. (2022)
	Coating technology	Tio2 nanotubes	Enhance alkaline phosphatase activity	Zhu et al. (2021)

Traditional Chinese medicine (TCM) formulas offer a safe and effective alternative for osteoporosis treatment, leveraging innovative dosage forms and synergistic effects of active ingredients. Qin et al. (2008) demonstrated that icariin in Erxian Decoction (EXD) exerts bidirectional regulation on bone metabolism in Ovariectomized (OVX) rats, promoting bone formation while inhibiting resorption. Additionally, Ren M. et al. (2023) confirmed that serum-derived icariin from EXD enhances Collagen Type I and Bone Morphogenetic Protein-2 (BMP-2) expression in osteoblasts by suppressing BK channels.

Regarding traditional Chinese patent medicines and simple preparations, Wang et al. (2025) demonstrated that the novel Kunxinning formulation alleviates bone loss by targeting perimenopausal estrogen deficiency *via* ICA-mediated CYP19A1 activation, with confirmed clinical safety. Zhu et al. (2012) established that Xianling Gubao Capsules safely increase lumbar spine bone mineral density (BMD) in postmenopausal women. Using network pharmacology and molecular docking, Wang et al. (2024) verified Gushukang’s inhibition of RANKL-induced osteoclast differentiation, downregulation of PTGS2 expression, and reduction of TRAP activity. Feng et al. (2022) utilizing 1H-NMR metabolomics, revealed that Gu-Gu-Dan ameliorates kidney yang deficiency syndrome through arginine metabolism, supporting the “kidney governs bone” theory. Li et al. (2022) further showed that Gushukang activates the IGF-1 pathway, reversing dexamethasone-induced muscle atrophy and bone loss in mice through bone-muscle crosstalk protection.

In summary, current research on traditional Chinese patent medicines and simple preparations has enhanced the bioavailability of icariin and other active constituents. These formulations demonstrate efficacy against osteoporosis through bone metabolism pathway regulation. Future studies should focus on: elucidating multi-component synergistic mechanisms, optimizing sustained-release delivery systems, and advancing large-scale clinical trials to overcome limitations of current dosage forms—notably transient efficacy, long-term instability, and dose-dependent effects.

### 3.2 Biomaterial design

As a natural anti-inflammatory flavonoid (Singh and Singh, 2025; Seyedi et al., 2023), ICA’s application in osteoporosis therapy is limited by the low bioavailability and poor targeting of conventional formulations (Zhao and Zhang, 2025). In biomaterials research, innovative delivery systems enable spatio-temporal controlled release, bone microenvironment modulation, and multi-mechanism synergy of ICA. These approaches address three critical aspects: local drug enrichment, inflammation-osteogenesis imbalance correction, and bone metabolic microenvironment remodeling—effectively overcoming the off-target effects and inefficiency of systemic administration.

To overcome ICA delivery bottlenecks, researchers have systematically investigated material structures and release mechanisms. Hydrogel systems have emerged as a primary focus: Zhang et al. (2025) find a composite hydrogel mimicking bone matrix properties for bone defect repair, though its mechanical strength/sustained-release efficiency balance requires optimization. 3D printing technology enables complex bone reconstruction: Kong et al. (2025) confirm strontium-substituted mesoporous bioactive glass/polycaprolactone scaffolds with uniform pores (achieved *via* electrospray printing), co-releasing Sr^2+^ and ICA to simultaneously activate osteogenic differentiation and suppress osteoclast activity; Wang W. et al., 2022) find layered titanium alloy scaffolds for controlled ICA/Mg^2+^ release, inducing macrophage M2 polarization and enhancing osseointegration. Photoresponsive innovations enable precise modulation: Li et al. (2024a) constructed selenium-gold multishell nanocomposites that trigger Se/ICA release under NIR irradiation, dually suppressing reactive oxygen species (ROS) and inflammatory factors; Yan et al. (2022) integrated upconversion nanoparticles with MMP13-sensitive peptides to link real-time osteogenesis monitoring with therapeutic feedback. Coating technology advances are significant: Zhu et al. (2021) co-loaded Sr/ICA onto TiO_2_ nanotube coatings, demonstrating enhanced alkaline phosphatase (ALP) activity and new bone formation in osteoporotic rats, accelerating clinical translation of titanium implants.

As research advances, scholars recognize that single delivery systems inadequately address OP’s multi-pathological mechanisms, driving exploration of multi-component synergy. Targeted delivery systems represent a key direction: Luo et al. (2024) confirm hydroxyapatite-responsive macrocyclic amphiphiles to enhance bone density *via* localized drug enrichment. Li et al., 2024c) find self-assembled ICA/Ca^2+^/zoledronic acid nanocomposites that release drugs under *in vitro* shock waves, reversing osteogenic-adipogenic differentiation imbalance. Immune microenvironment regulation proves critical: Chai et al. (2022) induced macrophage M2 polarization and bone resorption inhibition using sulfonated polyetheretherketone; Mosqueira et al. (2020) demonstrated strontium/ICA co-loaded bioactive glass microspheres reverse diminished osteogenic potential in osteoporotic stem cells; Lu et al. (2021) confirm PLGA-co-encapsulated quercetin/curcumin nanoparticles inhibiting trabecular bone loss by enhancing flavonoid bioavailability. Han et al. (2025) revealed Epimedium-derived extracellular vesicles promote osteodifferentiation *via* the PI3K/Akt/mTOR pathway, with composite bone repair materials functioning as dual-action “phytoestrogen carriers.”

Current studies demonstrate that novel ICA biomaterials substantially enhance therapeutic precision and efficacy for osteoporosis through intelligent responsive release, ionic synergy, and immune modulation. However, key challenges requiring resolution include: biocompatibility and metabolic safety verification, and optimization of mechanical properties *versus* drug release kinetics.

## 4 Advances in the application of animal models in the anti-osteoporotic effects of icariin

### 4.1 Model of postmenopausal osteoporosis

Ovariectomized (OVX) rats, serving as a classic model for mimicking postmenopausal osteoporosis (Wang et al., 2021; Chen et al., 2021), offer a pivotal platform for investigating the bone-protective mechanisms of ICA. Recent research has confirmed that, in this model, ICA not only mimics the osteogenic effects of estrogen but also exhibits non-estrogen-dependent mechanisms, such as modulating neuropeptide signaling and stabilizing bone mineral phase structures, thereby providing a crucial theoretical foundation for the development of novel anti-osteoporosis drugs (as shown in Table 2).

**TABLE 2:** Progress in the treatment of osteoporosis animal models with icariin.

Large category	Classification	Model introduction	Key efficacy indicators	Administration	Proposed mechanism	References
Rodent	Postmenopausal	OVX	• Vertebral BMD ↑∼1.9% • Serum activity of E2 ↑ significantly	Oral; 125 mg/kg/day ICA	Regulation of estrogen receptor pathways	Li et al. (2014)
		OVX + iron dextran-induced iron overload	• BMD, BV/TV, Tb.N ↑ significantly • SF ↓ significantly • Osteogenic Markers ↑ (ALP, OC, OPG). Significantly	Oral; 5–20 mg/kg/day icaritin (IT) for 8 weeks	• Promotes Osteogenesis by increasing osteogenic markers • Reverses Iron Overload by chelating labile plasma iron via complexation at the 3-OH site, forming an ICT-Fe(III) complex	Jiang et al. (2023)
		OVX-induced postmenopausal fracture	• Femoral BMD ↑ significantly • Tb.N ↑ significantly	Oral; 600 mg/kg/day ICA	Regulation of the OPG/RANKL pathway	Zhang et al. (2020)
	Glucocorticoids	Prednisolone implantation	• BV/TV ↑ ∼11.3% • Tb.Th ↑ ∼10.3% • BMD ↑ significantly • ALP activity ↑ significantly	Oral; 250 mg/kg/day ICA	Modulates the PI3K/Akt/GSK3β/β-catenin integrated signaling pathway	Hu et al. (2017)
		Prednisolone injection	• BV/TV, Tb.N ↑ significantly • BMD ↑ significantly		Regulating the balance of the EphB4/Ephrin-B2 pathway	Huang M. et al. (2020)
	Old Aged	Aged mice (18-month-old)	• Tibia BV/TV ↑ significantly • Femur maximal load ↑ ∼ 22%, elastic modulus ↑ ∼ 45% • Net calcium retention ↑ significantly	Oral; 0.38 g/kg/day Herbal formula Gushukang	• Stimulates vitamin D production (↑ 25(OH)D and 1,25(OH)2D) • Improves calcium balance by regulating transporters (↑ TRPV5/6, ↓ Claudin-14)	Li et al. (2020)
	Diabetes	Streptozotocin (STZ) injection	• Blood glucose ↓ from 417 to 98 mg/dL • Lumbar & Femur BMD ↑ significantly • BV/TV ↑, Tb.Th ↑, Tb.Sp ↓ • Bone turnover markers (CTX-1, ALP, etc.) ↓ • Bone marrow adipocyte density ↓	Oral; 100 mg/kg/day for 8 weeks	• Reduces blood glucose. • Inhibits bone turnover. • Suppresses bone marrow adipogenesis. • Upregulates osteogenic signaling: ↑ RUNX2, ↑ OPG/RANKL ratio.	Qi et al. (2019)
Nonrodent species	Chicken	Caged layer osteoporosis (CLO) in older hens	• Femur BMD ↑ 49.3% • Tibia BMD ↑ 38.9% • Improved bone microstructure • Enhanced serum antioxidant capacity	Dietary supplementation; 0.5–2.0 g/kg/day feed	Regulates key metabolic pathways • Pyrimidine metabolism • Taurine and hypotaurine metabolism • Glycerophospholipid metabolism	Huang J. et al. (2020)
		Tibial dyschondroplasia (TD) induced by thiram	• Significantly reduced average TD score • Increased vascular area in the growth plate • Upregulated BMP-2 expression	Drinking water; 10 mg/kg/day for 10 days	Promotes growth plate vascularization and Cartilage repair by up-regulating BMP-2 signaling pathway.	Iqbal et al. (2018)
	Fish	Glucocorticoid- induced: prednisolone (PNSL)-induced bone formation inhibition in zebrafish larvae	• SSA, COD ↑ • Bone Mineral Content ↑ (Ca, K, Mg, Zn, Fe) • Locomotion ↑ (Moving Distance, Average Speed, Travel Frequency)	• Water exposure; ICA, 0.1, 1.0, or 10.0 μM ICA +25 μM PNSL • Water exposure; IT, 0.1, 1.0, or 10.0 μM IT + 25 μM PNSL	• Inhibiting osteoclast differentiation via the OPG/RANKL/RANK pathway • Promoting osteoblast differentiation and bone formation via the BMP and Runx2 signaling pathways.	Jiang J. et al. (2022)
		Iron overload-induced: ferric ammonium citrate (FAC)-induced osteoporosis in zebrafish larvae	• Bone Mineralized Area ↑ • IOD ↑ • Locomotion ↑	Water exposure; 0.1, 1.0, 10.0 µM IT	Reverses iron overload-induced bone loss and behavioral deficits by chelating excess iron and promoting osteogenesis.	Jiang et al. (2023)
		Inflammation-induced: CuSO4-induced and tail-cutting-induced inflammation in zebrafish larvae	• Macrophage Migration ↓ • ROS Generation ↓ • Locomotor Impairment ↓ • Inflammatory Cytokines ↓ (il-1β, il-6, il-8, tnf-a, etc.)	Water exposure; 80, 160, 320 μM EB	Regulating the MAPK/NF-κB/NOD-like receptor signalling pathways	Liu L. et al. (2024)

Abbreviations: OVX, Ovariectomy; BMD, Bone Mineral Density; E_2_17β-estradiol; BV/TV, Bone Volume/Tissue Volume; Tb.N, Trabecular Number; SF, Serum Ferritin; ALP, Alkaline phosphatase; BMP-2, Bone morphogenetic protein-2; SSA, Skeleton Stained Area; COD, Cumulative Optical Density; IOD, Integral Optical Density.

In recent years, significant advancements have been achieved in elucidating the bone-protective mechanisms of ICA in OVX rat models. Li et al. (2014) demonstrated that ICA exerts a dual role in OVX rats: promoting new bone formation, inhibiting bone resorption, and suppressing bone marrow adipogenesis. Yang et al. (2014), through comparative studies on OVX rats, confirmed that although icariin exhibits a slightly lesser bone-protective effect compared to genistein, both can mimic estrogenic actions, significantly enhance BMD, and hold potential for osteoporosis treatment. Liu Y. et al., 2017) conducted a meta-analysis that synthesized evidence to show that ICA notably increases BMD and improves bone microstructure in OVX rats. Zhang et al. (2007) reported that ICA-derived phytoestrogens significantly delayed lumbar spine BMD loss in postmenopausal women at an advanced stage randomized controlled trial, without causing endometrial hyperplasia or estradiol fluctuations. Building upon these findings, Liu et al. (2012) discovered that the combination of ICA and exercise therapy enhances the bone biomechanical properties of OVX rats and upregulates the expression of Osterix, a key osteoblast gene. Further mechanistic investigations by Ouyang et al. (2014) revealed that a compound extract containing ICA (*Bushen Huayu* extract) may inhibit IL-6 expression, increase osteoblast counts, and decrease osteoclast numbers by modulating estradiol levels. Zhao et al. (2016) significantly improved the trabecular bone microstructure in OVX rats by upregulating osteoprotegerin and inhibiting the expression of receptor activator of RANKL.

It is noteworthy that the mechanism of action of icariin extends beyond direct regulation of bone metabolism. Liu et al. (2018) innovatively demonstrated its role in regulating the “brain-spinal cord-bone axis” by upregulating NPY1R/CRLR and other factors in bone tissue. In addressing the clinical challenge of OP fractures, Zhang et al. (2020) verified that ICA facilitates fracture healing by activating the OPG/RANKL pathway,and there are no uterine stimulation side effects.

In summary, ICA exerts anti-osteoporotic effects in OVX rats through a triple mechanism: direct regulation of bone metabolism; intervention in lipid-bone balance; and modulation of the neuropeptide axis. Its therapeutic efficacy remains effective and safe in complex scenarios including fracture repair and microgravity-induced bone loss. However, current research exhibits significant limitations: mechanisms are primarily validated at the tissue level with unclear cellular specificity, and the clinical translation evidence chain requires completion through standardized studies.

### 4.2 Glucocorticoid-induced osteoporosis model

The underlying pathology of glucocorticoid-induced osteoporosis (GIOP) is intricate (Chen M. et al., 2023; Wang et al., 2019), with ICA demonstrating distinctive advantages in regulating bone metabolism through multiple targets. Animal studies have validated its efficacy in enhancing trabecular bone structure, preserving bone cell viability, and maintaining calcium homeostasis.

During GIOP pathogenesis, icariin regulates bone metabolic balance *via* multi-target signaling pathways. Hu et al. (2017) demonstrated using *in vitro* and in vivomodels that ICA activates the ERK pathway to inhibit osteocyte apoptosis and attenuate bone loss. Further studies reveal icariin’s regulatory effects on osteoblast differentiation: Lin et al. (2017) identified ICA-mediated targeted suppression of the GILZ pathway, reversing dexamethasone-induced osteogenesis inhibition and restoring mineralization capacity. Notably, ICA’s anti-resorptive effects involve microRNA regulation—Ma et al. (2018) confirmed ICA activates microRNA-186 to suppress cathepsin K expression, thereby reversing hyperactive bone resorption and structural deterioration. In GIOP mouse models, Zhang et al. (2015) observed ICA-enhanced bone formation markers, suppressed resorption markers, improved calcium homeostasis, and restored bone microarchitecture. Beyond classical pathways, icariin’s bidirectional modulation of the EphB4/Ephrin-B2 axis provides new evidence for its polypharmacology: Huang M. et al. (2020) and Qiu et al. (2023) demonstrated ICA and its analog Epimedin C reverse trabecular loss and rebalance bone formation-resorption through this axis. Morphologically, Yang et al. (2023) utilized synchrotron radiation imaging to confirm ICA significantly repairs early GIOP-associated femoral head necrosis lesions and reconstructs trabecular networks.

In summary, ICA enhances GIOP through a synergistic effect involving multidimensional mechanisms. Its homolog, Epimedin C, exhibits the ability to bolster bone metabolism homeostasis by modulating Runx2/LGR4 expression, hinting at the potential existence of shared targets among icariin-like compounds. Nevertheless, current research is confined to preclinical models, and the interplay among various icariin pathways remains unelucidated. Moving forward, it is imperative to develop animal models that more accurately mirror the pathological features of human GIOP to validate its therapeutic efficacy.

### 4.3 Elderly osteoporosis model

The elderly osteoporosis model is characterized by aging-induced reductions in bone formation and disrupted bone turnover, culminating in low bone mass (Johnston and Dagar, 2020; Qadir et al., 2020). As a core anti-osteoporotic agent, ICA demonstrates potential for improving bone density quality in aged models including caged laying hens (CLO), SAMP6 mice, and senile osteoporosis (SOP) rats. Its mechanisms involve regulating osteogenesis-related genes, modulating signaling pathways, and restoring calcium homeostasis.

Research on ICA’s anti-osteoporotic mechanisms in aged models has progressively revealed its metabolic and molecular regulatory networks through multispecies studies. Huang J. et al. (2020) demonstrated *via* metabolomics that ICA improves bone microarchitecture and homeostasis in CLO by modulating pyrimidine/taurine/lipid metabolism. Building on this, Xu et al. (2018) focused on osteoblast differentiation in senescence-accelerated SAMP6 mice, revealing ICA enhances BMP-2-driven osteogenesis through connective tissue growth factor downregulation. As mechanistic understanding deepens, research has expanded to ICA-containing compound preparations: Li et al. (2020) developed Gushukang (ICA-containing traditional herbal formula) that synergistically activates vitamin D metabolism, bidirectionally regulates calcium transporters, and improves bone structure/mechanical properties plus calcium homeostasis in high-calcium-diet aged mice. Separately, Li et al. (2024b) established that an Epimedium-Ligustrum lucidum compound containing ICA ameliorates bone microstructure and oxidative damage in SOP rats by balancing autophagy-apoptosis equilibrium *via* the PI3K/Akt/mTOR axis.

In summary, the multi-target mechanisms of ICA and its compounds have been elucidated through metabolic, differentiation-regulatory, and compound optimization perspectives. Limitations include: heterogeneity in mechanisms explaining pathological variations across animal models; and unclear multi-component synergistic mechanisms.

### 4.4 Diabetes osteoporosis model

Diabetic osteoporosis (DOP) is characterized by hyperglycemia-induced bone turnover imbalance, ROS accumulation, and bone marrow adiposity, coupled with OPG/RANKL signaling dysregulation and primary cilia/Gli2 pathway inhibition (Kupai et al., 2024; Romero-Díaz et al., 2021). When investigating ICA’s intervention mechanisms in DOP, studies reveal its multi-target regulatory properties across diverse models.

Qi et al. (2019) demonstrated in STZ-induced rats that ICA lowers blood glucose, inhibits marrow steatosis, increases bone density, and reduces bone turnover markers. Zhang et al. (2020) confirmed *via* proteomics that Zishen Jiangtang Pill, a compound containing ICA, improves bone microstructure in DOP rats by modulating ribosome pathways and vitamin/fat metabolism. Notably, Liu et al. (2022) revealed that ICA promotes osteogenic differentiation by clearing ROS to maintain mitochondrial primary cilium homeostasis, thereby activating the primary cilium/Gli2/osteocalcin (OCN) pathway.

In summary, ICA effectively reverses bone loss through dual regulation of bone metabolism and ROS clearance. Future efforts should focus on advancing its clinical translation and further elucidating its multi-target mechanisms, aiming to develop a natural, multi-target anti-osteoporosis agent that integrates blood glucose reduction, bone protection, and antioxidant functions.

### 4.5 Other animal species models

The expansion of osteoporosis research into non-mammalian models offers new perspectives for studying ICA’s anti-osteoporotic mechanisms. Aquatic models, such as zebrafish and bluefish, leverage their genetic and high-throughput advantages to provide insights into ICA’s action and help analyze the impact of glycosylation modifications on its activity (Carnovali et al., 2020; Priya et al., 2023). Similarly, chicken models have demonstrated therapeutic efficacy in studies of avian bone diseases (Sarnella et al., 2024; Dodgson, 2007).

Recent studies highlight the unique advantages of non-rodent models in elucidating ICA’s anti-osteoporotic mechanisms. Pham Pham et al. (2021) innovatively applied the I-M quantification method in a bluefish RANKL model, demonstrating that ICA provides bone protection equivalent to alendronate sodium. In zebrafish models, multi-pathway mechanisms of ICA have been revealed: Jiang J. et al. (2022) verified through molecular docking and zebrafish assays that ICA and icaritin (IT) effectively reverses bone injury. Notably, Jiang et al. (2023) further confirmed using zebrafish and rat models that IT promotes bone formation by chelating iron ions to reverse overload. This mechanistic diversity extends to inflammation-related osteoporosis, where Liu L. et al., 2024) found that icariin B (EB) inhibits neuroinflammation and ROS accumulation in zebrafish by regulating the MAPK/NF-κB/NOD-like receptor pathway.

Huang J. et al. (2020) demonstrated that dietary ICA supplementation in aged caged chickens effectively increases BMD and alleviates osteoporosis symptoms. These beneficial effects are attributed to ICA’s precise modulation of endogenous pyrimidine, taurine, and lipid metabolism. Notably, ICA’s osteoreparative capacity extends beyond aged bone: Iqbal et al. (2018) successfully repaired tibial dyschondroplasia in fast-growing chickens by activating the BMP-2 signaling pathway.

In summary, non-mammalian models effectively elucidate the complex multi-target/pathway mechanisms through which ICA and its derivatives exert bone-protective effects. Future research should prioritize cross-species mechanism validation, methodological standardization, and optimization of active structural components to accelerate ICA’s translational pipeline for osteoporosis therapy.

## 5 Molecular mechanism of icariin against osteoporosis

### 5.1 Osteogenesis and osteoclast regulation

The homeostatic imbalance between osteoblast-mediated bone formation and osteoclast-driven bone resorption constitutes a fundamental pathological mechanism in osteoporosis. Therapeutic strategies therefore focus on restoring bone metabolic equilibrium by modulating these cellular activities (Hou and Tian, 2022; Zhang X. et al., 2023). As a natural anti-osteoporotic agent, ICA exhibits systematically validated multi-target effects (Xue et al., 2016): it inhibits osteoclast differentiation and bone resorption (Cao et al., 2012; Fang et al., 2017; Liang et al., 2012) while promoting osteogenesis through activation of bone-forming pathways (Meng et al., 2005; Tang et al., 2018). This bidirectional regulatory capacity provides a mechanistic basis for its clinical translation (as shown in Table 3).

**TABLE 3:** Progress in molecular mechanism of icariin treatment of osteoporosis.

Large category	Classification	Specific content	Effect	References
Mechanism	Osteogenesis and osteoclast regulation	BMP/Wnt pathway	Promote osteogenic differentiation	Li et al. (2016)
		BMP-2/Runx2 pathway		Saud et al. (2019)
		RhoA TAZ signaling pathway	Promote proliferation of osteoblasts	Ye et al. (2017)
		RANKL induced pathway	Inhibit osteoclast differentiation	Cheng et al. (2022), Yang et al. (2024), Xu Q. et al. (2019), Wang Q. et al. (2018)
	Regulation of adipogenic differentiation	Notch signal	Promote adipogenesis	Liu H. et al. (2017)
		Wnt pathway	Inhibit fat production	Chen X. et al. (2025)
		PPARγ/C/EBP/FABP4	Regulating the proliferation and differentiation of adipocytes	Liu Y. et al. (2017)
	Regulation of autophagy and apoptosis balance	Bcl-2/Beclin1/LC3-II	Induce autophagy	Li et al. (2024b)
		AMPK/ULK1 Akt/mTOR/ULK1 axis	Reverse autophagy disorder	Zou et al. (2024)
		TNF -αsignaling	Activate autophagy	Bai et al. (2023)
		PI3K/Akt/mTOR		Chen et al. (2022)
	Anti-inflammatory mechanism	MAPK like receptor pathway	Inhibit inflammation	Liu W. et al. (2024), Song et al. (2024), Zhang et al. (2022), Hsieh et al. (2011)
		NF-κB like signaling pathway	Reduce pro-inflammatory factors	Liu L. et al. (2024), Hsieh et al. (2011), Hwang et al. (2018)
	Iron overload intervention mechanism	ERK1/2/JNK-MAPK	Resist mitochondrial damage induced by iron overload	Yao et al. (2019)
		PI3K/AKT/mTOR		
	Anti oxidative stress mechanism	RANKL induction	Inhibit the production of reactive oxygen species	Ji et al. (2022)
	Regulation of estrogen signaling pathway	ER receptor	Inhibit RANKL induced osteoclasts and promote osteogenesis	Sun et al. (2021), Zhang et al. (2012)
	Gut microbiota bone axis regulation	RANKL/OPG pathway	Enhance metabolism and improve bone microstructure	Wang W. et al. (2022)
	Expression and functional regulation of osteocalcin	FABP4	Promote osteogenesis	Zhao et al. (2024)
	Regulation of alkaline phosphatase activity	BMP-2/β-catenin	Promote mineralization	Oh et al. (2019)

Significant advances have been made in understanding ICA’s molecular regulation of osteogenic differentiation. Notably, Li et al. (2016) demonstrated in OVX mice that the ICA-ferulic acid combination synergistically activates BMP/Wnt signaling, enhances bone matrix protein expression, and increases bone density/mineralization. Ye et al. (2017) established that ICA promotes proliferation and osteogenic differentiation in rat adipose-derived stem cells through RhoA-TAZ signaling, with this effect being abolished by RhoA inhibitor C3. Jin Y. et al. (2024) revealed ICA upregulates METTL14-mediated P4HB m^6^A modification to enhance stability and bone formation in BMSCs. Chen et al. (2007) identified cell-type specificity: ICA enhances osteogenic differentiation in rat BMSCs but shows no significant effect on rat calvarial osteoblasts. Clinical translation studies further validate ICA’s therapeutic potential. Saud et al. (2019) systematically reviewed ICA’s regulation of mesenchymal stem cell fate *via* BMP-2/Runx2 and other pathways, supporting bone regeneration material development. Yang et al. (2019) emphasized that ICA’s multidimensional control of BMSC proliferation/differentiation constitutes a key anti-osteoporotic mechanism. Corroborating this, Wang Z. et al. (2018) demonstrated ICA’s dual regulatory function: activating BMSC osteogenesis while inhibiting osteoclast activity.

The mechanisms underlying ICA’s inhibition of osteoclasts have been progressively elucidated. Yang et al. (2024) demonstrated that ICA suppresses RANKL-induced osteoclast differentiation *via* the ERα/c-Src/RANK pathway. Notably, Ma et al. (2022) confirmed that its metabolite Baohuoside I (BS) inhibits osteoclast differentiation and bone resorption by blocking the MAPK/NF-κB pathway. Further mechanistic insight was provided by Xu Q. et al. (2019) who showed ICA synergistically inhibits RANKL-triggered NF-κB/MAPK signaling and disrupts F-actin ring formation, thereby impairing bone resorption. Through *in vitro* studies, Kim et al. (2018) revealed that ICA downregulates TRAF6 expression and ERK phosphorylation while reducing osteoclast-specific markers, offering novel therapeutic strategies for bone-related diseases. *In vivo* evidence confirms ICA’s osteoclast-inhibitory effects are linked to systemic bone homeostasis regulation: Cheng et al. (2022) reported in a thioacetamide-induced rat model that ICA reduces osteoclastogenesis through downregulation of RANKL-p38/ERK-NFATc1 signaling. Corroborating this, Wang Q. et al. (2018) demonstrated in OVX mice that ICA combined with mechanical loading increases the OPG/RANKL ratio while upregulating osteogenic genes and downregulating osteoclast-related genes.

**FIGURE 4:**
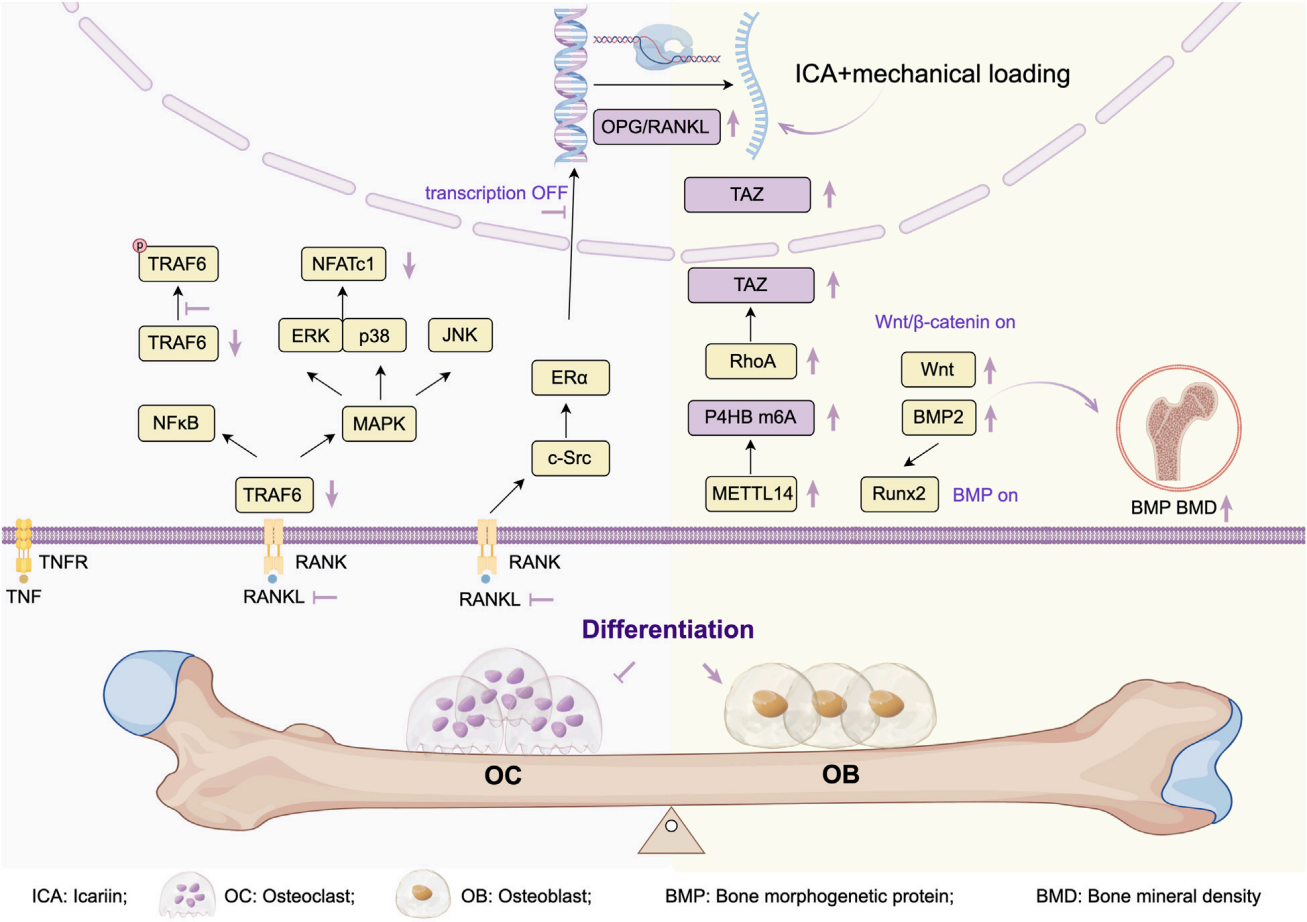
Research progress on ICA treats osteoporosis by regulating osteogenic and osteoclast activity.

In summary, ICA primarily inhibits osteoclast differentiation by modulating the RANKL signaling network, exerting anti-osteoporotic effects through: OPG/RANKL regulation, metabolic improvement, and enhanced bone coupling (Figure 4). Current research faces three key limitations: mechanisms predominantly validated in cellular/small-animal models lack clinical evidence; unclear bioactivity and synergy of ICA metabolites; insufficient understanding of osteoclast-BMSC crosstalk mechanisms.

### 5.2 Regulation of adipogenic differentiation

Excessive adipogenic differentiation and suppressed osteogenic differentiation of bone marrow mesenchymal stem cells (BMSCs) constitute key cellular mechanisms underlying bone marrow adiposity, bone mass reduction, and osteoporosis (An et al., 2023; Du et al., 2021). Recently, ICA has emerged as a natural active compound for osteoporosis treatment, regulating stem cell differentiation balance and bone metabolic microenvironment *via* multi-target therapy. Through stem cell transplantation and epigenetic strategies, ICA bidirectionally modulates osteogenic-adipogenic differentiation, reshapes bone-lipid homeostasis, and provides a theoretical basis for ICA-based biomaterials.

Key breakthroughs in ICA’s regulation of stem cell differentiation include: Lin et al. (2024) demonstrated ICA inhibits Hippo-YAP/TAZ phosphorylation, promoting osteogenesis while suppressing adipogenesis in Adipose-Derived Stem Cells (ADSCs). Notably, Chen X. et al. (2025) revealed in postmenopausal OP models that ICA enhances SOST promoter methylation to inhibit ERα binding, thereby upregulating Wnt signaling and driving BMSC osteogenic differentiation. This epigenetic mechanism was further confirmed by Li et al. (2024d) showing ICA blocks BMSC adipogenesis *via* S100A16 inhibition.

At the hormone signaling level, multiple studies highlight the pivotal role of estrogen receptor (ER) pathways. Li et al. (2018) confirmed ICA upregulates osteogenic markers while suppressing adipogenic factors in rat BMSCs *via* ER. Zhang et al. (2016) further elucidated ICA’s dual ER-mediated regulation: inhibiting osteoclast activation through OPG/RANKL upregulation and blocking osteoblast-to-adipocyte transdifferentiation. Notably, Liu H. et al. (2017) demonstrated ICA inhibits Notch signaling and downregulates PPARγ/C/EBPα/FABP4, suppressing BMSC adipogenesis in OVX rats. This discovery is similar to the clinical approach of Zhao et al. (2024) - ICA improves bone microstructure by inhibiting FABP4 expression and increasing OCN levels, confirming its ability to inhibit key factors in fat metabolism. Significantly, Zhang et al. (2017) established ICA’s bidirectional effects in tri-cell coculture systems: promoting MC3T3-E1 osteogenesis, inhibiting RAW264.7 osteoclastogenesis, and reducing BMSC adipogenesis.

**FIGURE 5:**
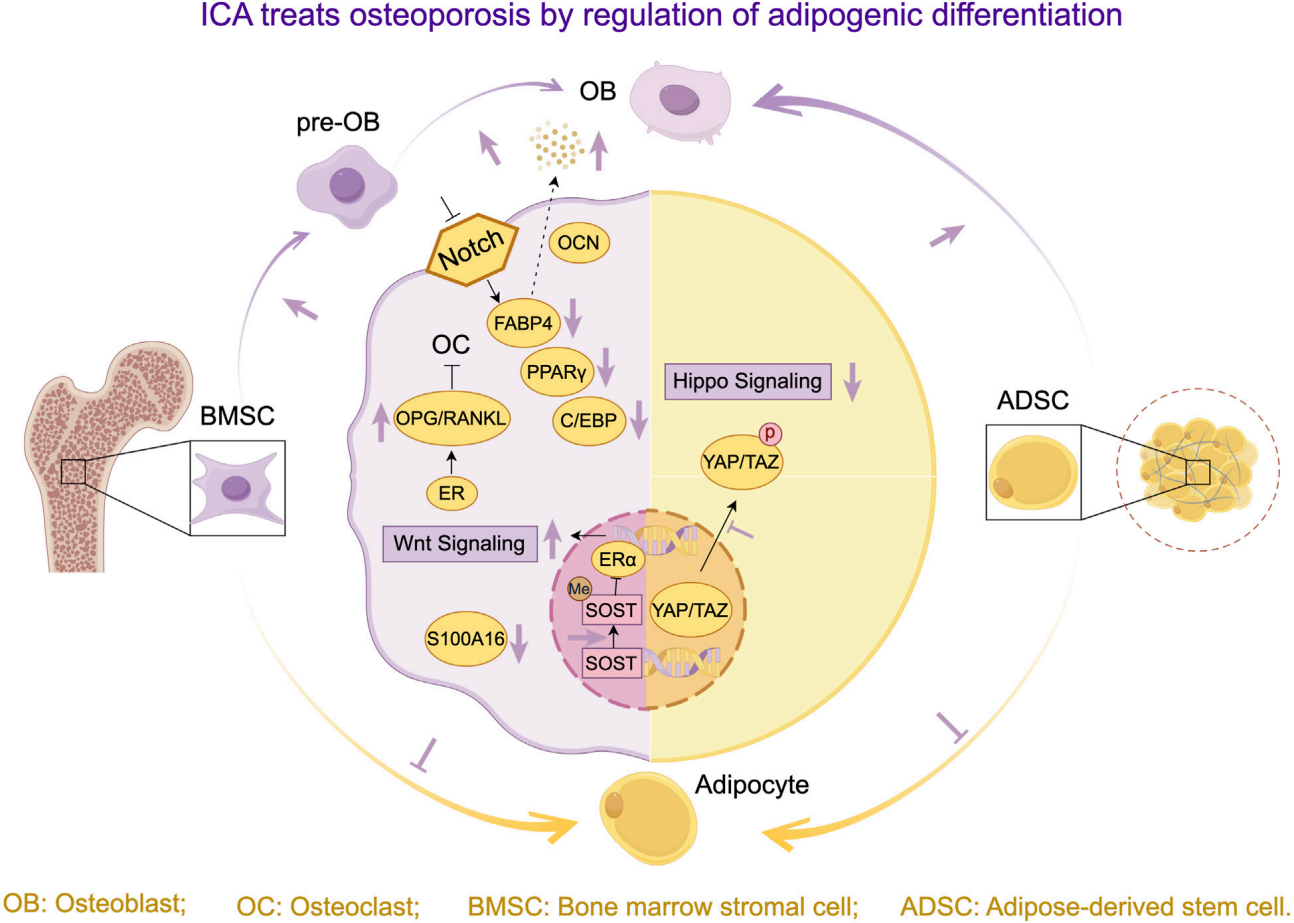
Research progress on ICA treats osteoporosis by regulating osteogenic and adipogenic differentiation.

In summary, ICA demonstrates advantages in stem cell transplantation, epigenetic modulation, and bone-lipid microenvironment remodeling by regulating osteogenic-adipogenic differentiation balance through multi-target synergistic pathways (Figure 5). Future research should focus on elucidating ICA’s polypharmacology—particularly osteogenic/adipogenic equilibrium—and exploring cross-pathway interactions to advance precision-targeted therapies.

### 5.3 Regulation of autophagy and apoptosis balance

Autophagy dysfunction compromises bone cell stress resistance, accelerates apoptosis, and disrupts bone remodeling balance, promoting osteoporosis development (Tang et al., 2019; Jin Z. et al., 2024). Osteoporosis therapeutic strategies are evolving toward multi-target synergistic regulation, with ICA emerging as a research focus due to its dual autophagy-apoptosis regulatory capacity (Liang et al., 2019; Verma et al., 2022; Shao et al., 2022).

The mechanisms underlying ICA’s regulation of autophagy and apoptosis are increasingly elucidated: Li et al. (2024b) demonstrated that an Epimedium-Ligustrum lucidum compound upregulates Bcl-2/Beclin-1/LC3-II while downregulating p53 in aged OP rats, ameliorating osteoblast apoptosis-autophagy imbalance. Zou et al. (2024) revealed ICA bidirectionally modulates autophagic flux *via* the AMPK/ULK1 and Akt/mTOR axes in OVX rats, reversing osteocyte autophagic dysfunction. This mechanism was confirmed by Liu W. et al. (2024) in ketogenic-diet OP models, where ICA inhibits mTOR phosphorylation, enhances autophagy, and reverses BMSC adipogenic-osteogenic imbalance. Notably, ICA’s polypharmacology extends beyond single pathways: Bai et al. (2023) established that ICA activates autophagy to inhibit TNF-α signaling and SASP secretion in senescent macrophages, rescuing osteogenic dysfunction in aged BMSCs. Chen et al. (2022) further corroborated in IL-1β-induced cartilage degeneration models that ICA alleviates degeneration by suppressing PI3K/Akt/mTOR phosphorylation and activating ULK1-mediated autophagy.

**FIGURE 6:**
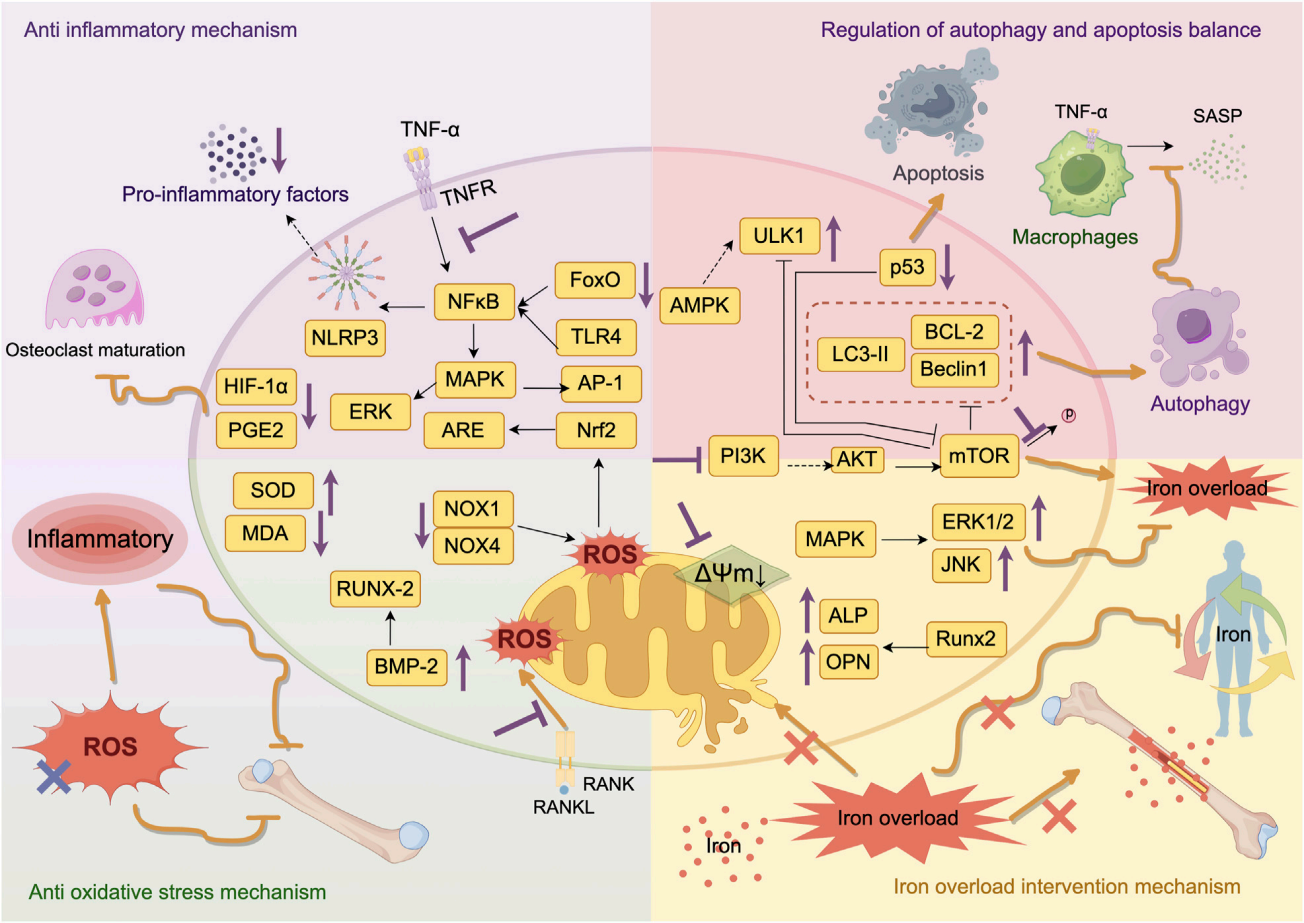
Research progress on the molecular mechanism of ICA in the treatment of osteoporosis by coordinating anti-inflammatory and antioxidant stress, regulating autophagy and apoptosis, and intervening in iron overload.

In summary, Epimedium and its active constituents induce osteoblast autophagy, suppress apoptosis, improve bone microarchitecture, and promote bone formation through key signaling pathway modulation (Figure 6). Future research should prioritize exploring targeted therapeutics that balance autophagy-apoptosis equilibrium for clinical osteoporosis treatment, with emphasis on optimizing multi-pathway synergistic regulation to enhance bone-targeting capacity and treatment efficacy.

### 5.4 Anti inflammatory mechanism

Inflammation activates osteoclasts and suppresses osteoblasts *via* pro-inflammatory cytokine release, disrupting bone remodeling equilibrium and accelerating osteoporosis progression (Lu et al., 2022; Xiang et al., 2025). Recently, the anti-inflammatory properties of ICA and its derivatives in OP treatment have garnered significant attention (Ma et al., 2020). Studies demonstrate that ICA exerts central therapeutic effects by synergistically reshaping the inflammatory microenvironment through key multi-pathway mechanisms.

ICA and its derivatives exhibit multidimensional anti-inflammatory effects: Liu L. et al. (2024) demonstrated in zebrafish models that EB attenuates inflammation by reducing ROS accumulation, modulating MAPK/NF-κB/NOD-like receptor pathways, and downregulating pro-inflammatory factors. Zhang et al. (2014) highlighted ICA’s synergistic regulation of bone cell inflammation and activation of osteoinductive pathways. As research progresses, ICA-regulated molecular networks are being clarified. Song et al. (2024) combined network pharmacology with experimental validation to show Epimedium components inhibit osteoclast markers/activity and bidirectionally regulate bone metabolism *via* FoxO/MAPK/TNF signaling. Zhang et al. (2022) established ICA’s ERK/MAPK-mediated estrogen-like effects suppress inflammation while promoting osteogenesis. In LPS models, Hsieh et al. (2011) confirmed ICA blocks osteoclast maturation and pro-inflammatory factors by inhibiting the MAPK/NF-κB/HIF-1α/PGE2 cascade. Hwang et al. (2018) extended findings to skin models, where ICA/ICT activates Nrf2/ARE while suppressing MAPK/AP-1/NF-κB, reducing MMP-1 and inflammatory cytokine release.

Collectively, ICA alleviates OP by targeting classical inflammatory pathways and cross-network systems to suppress inflammation, demonstrating pleiotropic effects in zebrafish and cellular models. Nevertheless, current evidence predominantly derives from *in vitro*/animal studies, and pathway crosstalk mechanisms within distinct inflammatory microenvironments require elucidation. Future research should focus on: identifying targets in aging-related chronic inflammation, and optimizing bone-targeted delivery strategies to accelerate clinical translation.

### 5.5 Iron overload intervention mechanism

Iron overload triggers excessive ROS generation *via* the Fenton reaction, inhibiting osteogenesis, promoting osteoclast differentiation, and disrupting bone homeostasis—a significant pathogenic factor in osteoporosis (Tian et al., 2016; Xia et al., 2019; Jiang Z. et al., 2022). ICA and its derivatives exhibit multifaceted intervention potential in both zebrafish and mammalian models.

The zebrafish model offers unique advantages for analyzing ICA components: Yan et al. (2025) demonstrated that ICA and its glycosides inhibit lipid peroxidation while enhancing osteogenic differentiation markers. Notably, Jing et al. (2019) elucidated mechanisms in iron-overloaded mice, showing ICA: inhibits mitochondrial membrane potential depolarization and ROS accumulation; restores Runx2/ALP/OPN expression in osteoblasts; suppresses osteoclast differentiation. *In vivo* analyses further revealed reduced bone marrow iron deposition and systemic iron metabolism regulation. Mechanistically, ICA’s cytoprotective effects involve mitochondrial homeostasis and signaling pathways: Yao et al. (2019) revealed ICA protects BMSCs against iron overload-induced mitochondrial damage by modulating ERK1/2/JNK-MAPK and PI3K/AKT/mTOR cascades.

In summary, ICA effectively mitigates iron overload-induced bone loss across diverse models (Figure 6). However, further exploration is warranted: pharmacological differences and structure-activity relationships between ICA and its glycosidic derivatives; crosstalk mechanisms between mitochondrial homeostasis and signaling pathways in iron-overloaded bone metabolism; clinical translational potential and long-term safety profiles.

### 5.6 Anti oxidative stress mechanism

Oxidative stress contributes to osteoporosis pathogenesis by damaging osteoblasts, promoting osteoclast activation, and accelerating bone metabolic imbalance (Zhang C. et al., 2023; Kimball et al., 2021; Iantomasi et al., 2023). Recent studies demonstrate ICA’s therapeutic potential for OP through multi-targeted regulation of oxidative stress equilibrium. It provides comprehensive intervention strategies *via* antioxidant effects, anti-inflammatory properties, and modulation of stem cell fate.

Recent studies have elucidated the pivotal role of ICA’s antioxidant mechanisms in osteoporosis treatment. Ji et al. (2022) demonstrated that ICA downregulates NOX1/NOX4, blocks RANKL-induced ROS production, and inhibits osteoclast differentiation—indicating its suppression of osteoclastogenesis *via* oxidative stress targeting. Xi et al. (2019) reported that Baohuoside I reduces serum pro-inflammatory cytokines, enhances SOD activity, and decreases Malondialdehyde (MDA) levels in OVX rats, promoting BMSC osteogenic differentiation through synergistic antioxidant and anti-inflammatory effects. Ma et al. (2014) further established that ICA inhibits ROS/MDA generation in osteoblasts while reducing inflammation and apoptosis, thereby maintaining RUNX-2/BMP-2 expression and protecting bone formation *via* phytoestrogen-like activity and anti-hypoxic effects.

Collectively, this evidence elucidates ICA’s molecular mechanisms for ameliorating osteoporosis through antioxidant pathways. Specifically, ICA targets the Cullin-3/Nrf2 axis to inhibit osteoclast differentiationand counter iron overload-induced lipid peroxidation (Figure 6). Nevertheless, current findings predominantly derive from animal/cellular models, necessitating validation of clinical translation potential *via* randomized controlled trials to advance precision osteoporosis therapeutics.

### 5.7 Regulation of bone metabolism homeostasis

ICA reshapes bone tissue energy metabolism homeostasis through precise modulation of estrogen signaling (Yao et al., 2025), gut microbiota (Zhang Y. et al., 2021), OCN (Mohammadi et al., 2024), and ALP (Omidvar et al., 2022), providing a targeted therapeutic strategy for metabolic disorder-associated osteoporosis (Xie et al., 2023; Zhang et al., 2011; Zhang et al., 2008).

#### 5.7.1 Regulation of estrogen signaling pathway

Estrogen deficiency, a primary etiology of osteoporosis, activates osteoclasts and disrupts bone resorption regulation, accelerating bone mass loss and microstructural deterioration (Yao et al., 2025; Kverka and Stepan, 2024; Wu W. et al., 2025; Zhu et al., 2024). Recent studies have progressively unveiled molecular mechanisms by which ICA modulates bone metabolism *via* ER stimulation (Wu Z. et al., 2017). Sun et al. (2021) demonstrated ICA upregulates the OPG/RANKL ratio and ALP/OPN activity through ER, enhancing osteoblast proliferation and mineralization. Luo et al. (2015) confirmed ICA restores osteogenic differentiation capacity of BMSCs in OVX rats *via* ER, progesterone receptor, and pS2 pathways. Song et al. (2013) revealed ICA activates ERK/JNK phosphorylation through ER, promoting proliferation and mineralization in MC3T3-E1 osteoblasts. Notably, Yang et al. (2013) established that ICA activates aromatase to stimulate estrogen synthesis, augmenting osteoblast ALP activity and OPG expression. This dual-regulatory mode shows clinical promise: Xue et al. (2012) found both ICA and estrogen improved bone density in OVX rats, but ICA uniquely inhibited osteoclastic resorption with significantly reduced uterine side effects. Complementarily, Zhang et al. (2012) delineated ICA and baohuoside-I suppress RANKL-induced osteoclast differentiation *via* ER-dependent pathways, decreasing TRAP and MMP-9 biomarkers. Mok et al. (2010) integrated mechanisms in OVX mice, showing ICA induces ligand-independent ERα Ser118 phosphorylation, upregulates OPG/RANKL mRNA ratio, and prevents bone loss without uterine hyperplasia.

#### 5.7.2 Gut microbiota bone axis regulation

Recent studies implicate gut-bone axis dysbiosis in osteoporosis pathogenesis *via* pro-inflammatory responses, immune dysregulation, and intestinal barrier impairment (Zhang Y. et al., 2021; Awuti et al., 2022; Zhang Y. et al., 2024). Integrating TCM with probiotics synergistically improves microbiota balance and enhances bioavailability. Intestinal microbiota-mediated metabolic mechanisms of ICA and its OP regulatory effects have emerged as research priorities (Fan et al., 2025). Wu et al. (2016) revealed key bacterial strains hydrolyze ICA into IT and desmethylicaritin, significantly increasing bioavailability and mediating estrogen-like anti-osteoporotic effects. However, pathological conditions may impair ICA’s metabolic efficiency. Zhou et al. (2015) observed reduced hydrolysis/absorption of 7-glucosylated flavonoids in OVX rats due to microbiota alterations, indicating diminished metabolic capacity under pathological states. Significantly, Wang S. et al., 2022) demonstrated ICA upregulates beneficial bacteria in OVX models, improving bone microarchitecture *via* microbial metabolite-mediated RANKL/OPG pathway regulation.

#### 5.7.3 Expression and functional regulation of osteocalcin

Elevated serum OCN levels significantly correlate with accelerated bone turnover in osteoporosis, serving as key biomarkers for diagnosing and monitoring postmenopausal osteoporosis (Mohammadi et al., 2024; Alam et al., 2019; Raymond et al., 1999). The mechanism by which ICA ameliorates osteoporosis through OCN pathway modulation has been systematically validated across multiple models (Chen et al., 2016). In postmenopausal osteoporosis models: Zhao et al. (2024) demonstrated ICA inhibits adipogenic marker FABP4, elevates serum OCN, and enhances trabecular bone structure Nian et al. (2009) found ICA reverses decreased serum OCN, restores BMD and biomechanical properties, with efficacy comparable to estrogen replacement therapy. Furthermore, He et al. (2018) extended this mechanism in simulated microgravity-induced bone loss models, showing ICA maintains OCN expression and enhances mineralization *via* stabilization of bone apatite crystals.

#### 5.7.4 Regulation of alkaline phosphatase activity

Elevated ALP levels serve as auxiliary indicators of accelerated bone turnover and active remodeling in osteoporosis (Omidvar et al., 2022; Chen R. et al., 2025; Che et al., 2023). Recent advances elucidate molecular mechanisms by which ICA regulates osteoblast differentiation and ALP activity: Xu et al. (2020) demonstrated ICA activates STAT3 phosphorylation, enhancing ALP activity and mineralization nodule formation in rat mandibular BMSCs; Xu Y. et al. (2019) further established that while ICA inhibits Notch signaling in MC3T3-E1/C3H10T1/2 cells, activation of this pathway abrogates ICA’s ALP-elevating effects.

The regulatory effects of ICA on ALP activity may vary across different cell models and experimental designs. Owen et al. (2020) observed in human embryonic stem cell-derived mesenchymal progenitors that although lithium chloride transiently increases ALP, it reduces ultimate mineralization—suggesting transient ALP elevation does not necessarily promote bone formation and requires evaluation alongside long-term mineralization outcomes. Complementary studies on natural products offer mechanistic insights for ICA: Oh et al. (2019) reported that brown algae PFF-A enhances ALP and mineralization of human BMSCs synchronously by activating BMP-2/β - catenin. Shi et al. (2022) in a systematic review, showing significantly lower serum ALP levels in patients receiving Epimedium monotherapy *versus* conventional drugs. This indicates Epimedium regulates bone metabolism by suppressing bone resorption markers with superior therapeutic efficacy.

In summary, ICA multi-targetedly regulates estrogen signaling, gut microbiota metabolism, OCN activity, and ALP function to reshape bone tissue energy metabolism homeostasis, offering novel targeted intervention strategies for metabolic disorder-associated osteoporosis. Future research should focus on: optimizing ICA metabolic efficiency under pathological conditions; elucidating multi-component synergistic mechanisms; and advancing clinical translation to achieve precise metabolic modulation of osteoporosis.

## 6 Summary, current challenges and future prospects

In summary, ICA-based formulations have significantly enhanced bioavailability and bone targeting through multi-component synergism in traditional preparations and biomaterial innovations, enabling precise local bone repair. Across diverse osteoporosis models (postmenopausal, glucocorticoid-induced, senile, and diabetic), ICA consistently improves bone microarchitecture and increases BMD. Its mechanisms encompass multidimensional regulation of bone-lipid homeostasis, neuropeptide axis modulation, and ROS scavenging. Mechanistic studies further reveal ICA promotes osteogenesis while suppressing osteoclastogenesis *via* multiple pathways, bidirectionally modulating BMSC osteogenic-adipogenic differentiation. Additional synergistic effects include restoring autophagy-apoptosis equilibrium, mitigating iron overload, exerting anti-inflammatory/antioxidant actions, and maintaining osseous microenvironment homeostasis.

Although icariin (ICA) shows unique advantages in the treatment of osteoporosis due to its multi-target mechanism, its research and clinical transformation still face several challenges. 1) Poor pharmacokinetic characteristics and delivery bottleneck: the oral bioavailability of ICA is low (about 12%), the plasma half-life is short (usually 1.2–3.5 h) (Hu et al., 2025), and the traditional dosage form is difficult to reach and maintain the effective therapeutic concentration in the bone tissue, which largely limits its curative effect. 2) The complex mechanism of action is not fully clarified: ICA plays a role through Wnt/β - Catenin, BMP, OPG/RANKL, MAPK and other signaling pathways, but the interaction network between the pathways and its core molecular targets are still unclear. In addition, many studies have found that the metabolites of ICA *in vivo* (such as IT, icaritin) also have biological activities, but the specific contribution of these metabolites and the synergistic mechanism with the mother drug are not enough. 3) Weak clinical evidence chain and transformation risk: at present, the vast majority of evidence supporting the efficacy of ICA comes from animal experiments (such as OVX rats, SAMP6 mice, *etc.*). Although some small-scale clinical studies (for example, Xianlinggubao capsule can safely increase the BMD of lumbar vertebra in postmenopausal women, and ICA derived phytoestrogen significantly delays the loss of lumbar BMD in postmenopausal women, without causing endometrial hyperplasia or estradiol fluctuations.) have shown positive effects, there is a lack of large-scale, multi center randomized controlled trials to systematically verify its long-term efficacy and safety in the population. In addition, the low bioavailability of ICA in human body may lead to its clinical efficacy less than expected.

In view of the above challenges and limitations, making full use of the advantages of ICA in the treatment of OP, future research should focus on: 1) optimizing targeted delivery systems: actively developing and optimizing new targeted delivery systems (such as smart hydrogels, 3D printing stents, nanoparticles), increasing the effective concentration and action time of ICA *in vivo*, in order to overcome the bottleneck of ICA bioavailability. 2) Deepen mechanism research: using multiomics technology and artificial intelligence, systematically analyze the multi-target action network of ICA, and clarify the contribution of its active metabolites, so as to explore and promote the combined treatment of ICA and standard drugs. 3) Promote clinical transformation and application: carry out more high-quality and standardized preclinical studies, promote well-designed clinical trials, determine the optimal dose scheme of ICA in human body, and monitor the long-term safety. At the same time, we should further explore the role of ICA in specific populations (such as diabetic osteoporosis) and its unclear molecular mechanism, and evaluate its synergistic effect with standard treatment (e.g., bisphosphonates), to expand its scope of indications.

## Abbreviations

OP, Osteoporosis; ICA, Icariin; BMSCs, bone marrow stromal cells; ROS, Reactive oxygen species; TCM, Traditional Chinese medicine; EXD, Erxian decoction; BMD, Bone mineral density; OVX, Ovariectomized; GIOP, Glucocorticoid-induced osteoporosis; CLO, Caged laying hens; SOP, Senile osteoporosis; DOP, Diabetic osteoporosis; IT, Icaritin; EB, Icariin B; BMSCs, Bone marrow stromal cells and Bone marrow mesenchymal stem cells; RANKL, nuclear factor-κB ligand; BMP-2, Bone morphogenetic protein-2; BS, Baohuoside I; MDA, Malondialdehyde; ER, Estrogen receptor; OOCN, steocalcin; ALP, Alkaline phosphatase; ADSCs, Adipose-derived stem cells.
